# How far can we go? A 20-year meta-analysis of dental implant survival rates

**DOI:** 10.1007/s00784-024-05929-3

**Published:** 2024-09-21

**Authors:** Johannes Raphael Kupka, Jochem König, Bilal Al-Nawas, Keyvan Sagheb, Eik Schiegnitz

**Affiliations:** 1 Department of Oral and Maxillofacial Surgery, Plastic Surgery, University Medical Center of the Johannes Gutenberg-University, Augustusplatz 2, 55131 Mainz, Germany; 2 Institute of Medical Biostatistics, Epidemiology and Informatics (IMBEI), University Medical Center of the Johannes Gutenberg University Mainz, 55131 Mainz, Germany

**Keywords:** Dental implants, Oral surgery, Retrospective studies, Prospective studies, Survival rate, Treatment outcome, Meta-analysis

## Abstract

**Objective:**

This meta-analysis aims to investigate the long-term survival rates of dental implants over a 20-year period, providing a practical guide for clinicians while identifying potential areas for future research.

**Materials and methods:**

Data were sourced from recent publications, focusing exclusively on screw-shaped titanium implants with a rough surface. Both retrospective and prospective studies were included to ensure an adequate sample size. A systematic electronic literature search was conducted in the databases: MEDLINE (PubMed), Cochrane, and Web of Science. The risk of bias for all studies was analyzed using a tool by Hoy et al.

**Results:**

Three prospective studies (*n* = 237 implants) revealed a mean implant survival rate of 92% (95% CI: 82% to 97%), decreasing to 78% (95% CI: 74%-82%) after imputation (*n* = 422 implants). A total of five retrospective studies (*n* = 1440 implants) showed a survival rate of 88% (95% CI: 78%-94%). Implant failure causes were multifactorial.

**Conclusion:**

This review consolidates 20-year dental implant survival data, reflecting a remarkable 4 out of 5 implants success rate. It emphasizes the need for long-term follow-up care, addressing multifactorial implant failure. Prioritizing quality standards is crucial to prevent overestimating treatment effectiveness due to potential statistical errors. While dental implantology boasts reliable therapies, there is still room for improvement, and additional high-quality studies are needed, particularly to evaluate implant success.

**Clinical relevance:**

Never before have the implant survival over 20 years been systematically analyzed in a meta-analysis. Although a long-term survival can be expected, follow-up is essential and shouldn't end after insertion or even after 10 years.

**Supplementary Information:**

The online version contains supplementary material available at 10.1007/s00784-024-05929-3.

## Introduction

Dental implantology has emerged as a cornerstone of modern dentistry and oral surgery [1]. Projections suggest that the prevalence of dental implants in the United States will soar to 23% by 2026 [2]. Considering this growth, questions regarding the long-term durability become increasingly pertinent. Extensive research reveals compelling evidence, demonstrating survival rates exceeding 90% even after ten years [3]. These findings are not only substantiated by many individual studies but also by comprehensive systematic reviews and high-quality meta-analyses [3–5].

It is worth noting that the difference between a 10-year and a 20-year lifespan has substantial implications for treatment planning [6]. If dental implants continue to exhibit such outstanding results over 20 years, it would necessitate a reevaluation of the decision-making process between preserving natural teeth (endodontics, periodontal therapy) and choosing implant insertion [6]. Attempting to delay implantation may carry the risk of additional infections and significantly complicate future implant-prosthetic treatments due to potential bone deficits [7]. In some cases, this could lead to a considerably costly reconstruction of prosthetic work for both the patient and, if applicable, the clinician. On the other hand, a systematic review concluded that prosthetic treatments on periodontally compromised teeth resulted in fewer complications compared to implant treatments [8]. It raises questions about how effective we truly are and how far we can go. Do dental implants genuinely offer a lifelong solution?

Furthermore, it should be noted that due to demographic changes, there is not only an achievement but also a demand for higher survival rates [2]. When assessing treatment alternatives, questions may arise about the feasibility of repeat operations in subsequent years, considering the patient's health [9]. However, if calculably high survival rates are consistently achieved over a 20-year period, it would significantly impact treatment approaches, offering many patients, even in advanced age, an improved quality of life through fixed prosthodontic care [10].

Previous meta-analyses were limited to a 10-year follow-up [3–5]. Additionally, advances in technology have transformed the characteristics of commercially available implants. The traditional implant with a machined surface is now rarely found and can no longer be described as state-of-the-art. Moreover, cylindrical and hollow-cylinder implants, although frequently included in earlier studies, have largely vanished from clinical practice [11, 12].

For these reasons, the aim of this systematic review with a meta-analysis was to assess the survival rate of screw-shaped dental implants with a rough surface after 20 years. This study seeks to provide a practical and realistic guide for clinicians while also identifying potential areas for future research and shedding light on any existing deficiencies.

## Methods

Considering the information mentioned in the Introduction, the following PICO criteria were defined:

- P—Patients over 18 years ("adults")
- I—Insertion of a screw-shaped dental implant with a rough surface
- C—No control intervention was recorded. The goal was to determine implant survival.
- O—20-year survival rate of dental implants

While conducting this systematic review, we adhered to the PRISMA guidelines and followed the corresponding checklist. The protocol was registered on PROSPERO (CRD42023402989).

### Inclusion and exclusion criteria

#### Study designs

In a first exploratory search, the number of prospective studies was considered too small to focus solely on them. Also, the reported data significantly differed in presentation and quality. For this reason, it was decided to include both prospective and retrospective studies. Observational as well as interventional studies were considered. Specifically, the following study types were included: Observational studies (prospective or retrospective cohort, case–control, cross-sectional and longitudinal studies), interventional studies (randomised and non-randomised controlled trials, controlled and uncontrolled trials).

Publications with less than 10 implants inserted were excluded. There were no restrictions regarding the publication date. The last search was conducted in February 2024, serving as the upper time limit. Only English-language publications were included.

#### Intervention

To increase relevance and realism, strict rules were established for the type of implant. They had to be screw-shaped implants made of titanium or a titanium alloy. The surface had to be rough (e.g., acid-etched, sandblasted, etc.). Obsolete or rarely used implant systems such as implants with a turned surface (e.g., Branemark), hollow screw, or hollow cylinder implants were excluded. Likewise, ceramic implants were not included.

The superstructure was divided in many studies into single crowns, fixed partial prostheses, fixed full-arch prostheses, and overdentures. The focus of this review is solely on the implant itself, and hence the types of restorations were recorded but not a basis for exclusion or inclusion. We excluded populations consisting solely of patients with severe conditions directly affecting bone regeneration, such as those on antiresorptive therapy or with osteoporosis. However, diabetes, for example, was not an exclusion criterion.

#### Setting

The study setting was not limited, allowing for a diverse range of environments such as university teaching hospitals, specialist dental practices, and general dental practices. This inclusive approach ensures that the results obtained reflect real-world scenarios and contribute to a more comprehensive understanding of the topic.

### Search strategy

A systematic electronic literature search was conducted in the databases: MEDLINE (PubMed), Cochrane, and Web of Science. The reference list and citations were also searched for relevant studies. There were no restrictions regarding the publication date to avoid missing any results. There were no restrictions regarding language during the search process, but only English language literature was included. Under these conditions, all subheadings, MeSH terms, as well as the title and abstract, were reviewed extensively following the strategy mentioned below. In addition, PROSPERO was thoroughly searched to identify any ongoing or recently completed systematic reviews.

The following terms were used for all databases with adapted subheadings and syntax. In the final step, the three issues were connected with “AND”.

- Complex 1: dental implants
  Dental implant*[MeSH Terms] OR tooth[Title/Abstract] OR teeth[Title/Abstract] OR dental[Title/Abstract] OR oral[Title/Abstract] OR implant*[Title/Abstract] OR osseointegrat*[Title/Abstract].
- Complex 2: exclusion of animal studies (inclusion of studies with animals AND humans)
  NOT (Animal*[MeSH Terms]) NOT (human*[MeSH Terms] AND Animal*[MeSH Terms]).
- Complex 3: twenty years of follow up
  20 NEAR year* [Title/Abstract] OR Twenty NEAR year* [Title/Abstract]

The search was documented using commercially available spreadsheet software (Microsoft Excel). Using the citation software Endnote 20, the results were collected, and duplicates and triplicates were excluded. Two of the authors (J.R.K. and E.S.) independently reviewed the results and selected suitable studies based on titles and abstracts. In case of discrepancies, a joint discussion was held to decide whether the study met the inclusion criteria. All authors re-examined the full texts for suitability, and authors were contacted in case of missing or incomplete data.

### Risk of Bias

J.R.K. assessed the risk of bias for all studies using a tool by Hoy et al., specifically tailored for prevalence studies, which was considered the most suitable in this case [13]. The results were reviewed and confirmed by all authors.

### Data

J.R.K. extracted the relevant data from the studies, and the other authors verified the results for accuracy. Any disagreements were resolved through joint discussions. Apart from outcome data, the names of authors, publication dates, and other study identification details were recorded, as well as the study type.

During data collection, it became apparent that certain assumptions had to be made for the studies to obtain data on implant survival: For controlled studies or those with multiple treatment groups, they were summed up and the resulting overall survival rate for the study group was calculated. Explanations are given in the results section for every study where necessary. Conversely, for studies where only one group was relevant for this review, only that group was included.

#### Imputation method

It is a well-known fact that particularly long-term studies have a high rate of patients or implants lost to follow-up. Therefore, an appropriate imputation method was chosen to obtain more realistic data for prospective studies. For this purpose, we relied on a publication by Akl et al. [14, 15], which recommends estimating the proportion of failed implants five times higher in the lost-to-follow-up (LTFU) group than in the group that could be tracked. This appears reasonable, especially considering that targeted follow-up increases implant survival and reduces the incidence of peri-implantitis. Nevertheless, this represents a conservative estimation that likely underestimates the actual survival rate. It should also be noted that this approach is supported by limited evidence and refers to a general procedure for follow-up data in medicine, not specifically for dental implantology. However, it is worth mentioning that Howe et al., in their meta-analysis of implant survival over 10 years, followed a similar approach [3].

#### Outcome measures

A complete case analysis was conducted for the primary evaluation of prospective studies. The analysis focused on implant survival rates rather than patients. For each study, the 95% confidence interval was individually calculated using the confint.binom function in R, from which the standard error (SE) was derived.

#### Secondary outcomes

The evaluation of retrospective studies did not require imputation since all studies used Kaplan–Meier curves, a more realistic method to handle implants that had no complete followed up. Consequently, the meta-analysis was conducted by gathering data on the survival rate, and if not provided, the 95% confidence interval was calculated as previously described.

#### Data synthesis

We utilized the statistical software environment R in conjunction with R Studio. The R package "metaprop" specializes in analyzing meta-analyses with binary data, particularly for proportions. It employs a random-effects model, the DerSimonian-Laird estimator, to account for heterogeneity among studies. This is necessary due to the inherent high heterogeneity expected. The study objectives vary significantly, as do applied methods, implant systems, patient characteristics, prosthetic restorations used, and others. The number of implant losses were calculated from extract proportions of losses and total number of implants. No adjustments were made for censoring and the fact that multiple implants per patients had been performed because papers did not provide sufficient information. Hence the precision is overestimated (confidence intervals are too small).

The results were graphically presented as forest plots, and a funnel plot was also generated to visualize publication bias. As a result, there were graphics for the complete case analysis of prospective studies, secondly for the dataset after imputation, and thirdly for the retrospective studies. The forest plots provide a visual representation of the combined effect sizes and their corresponding confidence intervals, allowing for an assessment of the overall impact of the interventions. The funnel plot aids in detecting potential publication bias, which can arise if studies with significant results are more likely to be published.

## Results

### Study selection

The initial database search across PubMed, Web of Science, and the Cochrane Library yielded a total of 805 results. After eliminating duplicates and triplicates, 621 unique records remained, which were subjected to a review of their titles and abstracts. Subsequently, 572 articles were excluded, and full texts were accessed for the remaining 49 articles. Following this stage, 8 articles (comprising 3 retrospective studies and 5 prospective studies) were deemed eligible for both qualitative and quantitative analyses.

**Fig. 1:**
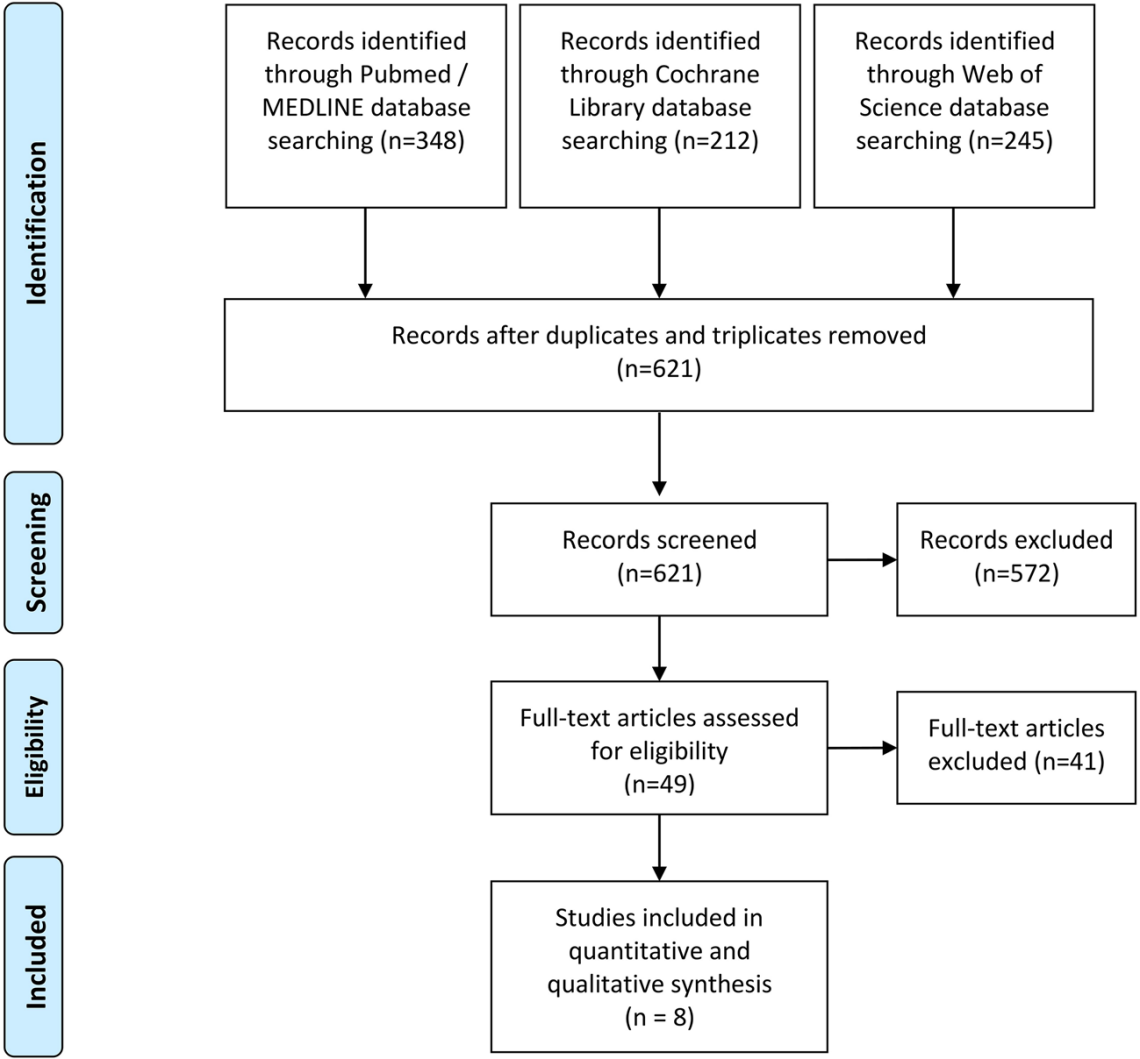
PRISMA flow diagram

The primary reasons for exclusion after a thorough review of full articles were: a follow-up period of less than 20 years (n = 11), the use of excluded implant systems (e.g., machined surface, outdated), or indistinguishable data from turned and rough surfaces (n = 18).Additionally, articles were excluded due to duplication with different titles (n = 3), as well as for being case reports or studies with small or specific study populations (e.g., Pappillon-Lévevre Syndrome) (n = 5). Furthermore, some articles were excluded due to their unavailability (n = 2), implausible data (n = 1) or not mentioned survival as outcome parameter (n = 1). A detailed PRISMA flowchart is provided in Fig. 1.

### Characteristics of the studies

The general characteristics of the studies are summarized in Table 1. Straumann and Astra Tech were the most commonly used implant systems [16–20]. Most studies were conducted in Europe, with Germany, Sweden, two from Belgium, and two from Italy represented [16, 18–22]. Additionally, there was one study from Asia (Japan) and one from America (USA) [17, 23]. Half of the studies were conducted in specialized practices, while the others took place in university centers. The retrospective studies exclusively comprised cohort studies, while the prospective studies had various designs, including a randomized controlled study [18], a prospective cohort study [20], and one with a split-mouth design [19].

**Table 1:** Study characteristics

	Study type	Setting	Country	Founded by industry	Implant system	Periodontitis	First included patient
Mangano et al. (2014)	Retrospective cohort study	Specialist practice	Italy	no funding	Mac System	Patients with history of periodical disease	1992
Becker et al. (2015)	Retrospective cohort study	University	Germany	no funding	Straumann	63,14% previously diagnosed periodontitis	1988
Horikawa et al. (2017)	Retrospective cohort study	7 private practices	Japan	no funding	Straumann	Not mentioned Some teeth were extracted due to periodontitis	1984
Cheng et al. (2022)	Retrospective cohort study	Specialist practice	USA	no funding	Bicon	Not mentioned	2000
Vrielinck et al. (2022)	Retrospective cohort stud	University	Belgium	no funding	(diverse)	Not mentioned	1998
Donati et al. (2018)	Randomized controlled clinical trial	University	Sweden	Astra Tech AB (Dentsply IH)	Astra Tech	moderate-to-advanced chronic periodontitis	1992
Jacobs et al. (2021**)**	Split mouth cohort study	University	Belgium	no funding	Astra Tech	Periodontal breakdown being the cause of tooth loss	1993
Roccuzzo et al. (2022)	Prospective cohort study	Specialist practice	Italy	no funding	Straumann	periodontally healthy patients, moderately, severely periodontally compromised patients	1998

The earliest patient data date back to 1984, and the most recent publications were in 2022. The study populations in the publications by Roccuzzo, Donati, Jacobs, and Mangano exclusively included patients with fixed prostheses [18–21]. Horikawa, Becker, Vrielinck, and Cheng also included patients with removable prostheses [16, 17, 22, 23]. Overall, the sample size (patients) ranged from 18 to 371, and the number of implants varied from 50 to 415. The rate of implants lost to follow-up in prospective investigations varied, ranging from just under 44% in Roccuzzo's study to 48% in Donati's and Jacobs' studies. The absolute number of implants and patients lost to follow-up (LTFU) was: Donati et al.: 36 implants, 26 patients; Jacobs et al.: 24 implants, 7 patients; Roccuzzo et al.: 125 implants, 65 patients [18–20]. The retrospective studies, however, did not disclose the number of patients lost to follow-up but instead compensated for this factor through Kaplan–Meier estimation.

### Risk of Bias

Since all studies exhibited a similar risk of bias, no weighting was applied in this regard (Table 2). Only one study received industry funding [18]. The other authors either declared no conflicts of interest or did not receive external funding.

**Table 2:**
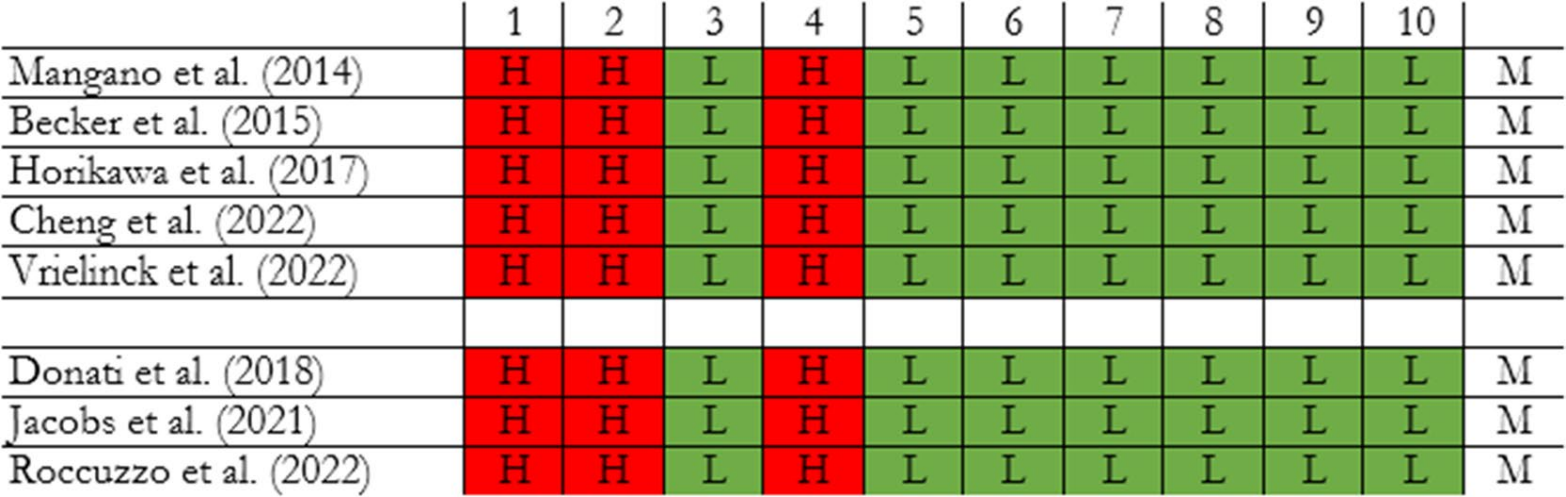
Risk of bias

Red (H) high risk of bias, White (M) Moderate risk of bias, Green (L) low risk of bias

### Summary of evidence quality across studies

The overall GRADE assessment indicated that the evidence quality across studies is considered very low [24]. Both prospective and retrospective studies were included, with a high selection bias expected, primarily due to the high rate of loss to follow-up. Detailed comments can be found in Table 3 and are not repeated here for clarity.

**Table 3:** Summary of evidence quality across studies (GRADE). LTFU: lost to follow up

Criteria for assessing quality of evidence of outcome	Comments	Quality of evidence
Risk of bias	5 of the 8 studies were retrospective cohort studies. Only one RCT was found. The LTFU-rate was high (over 43%) after 20 years	Very low
Inconsistency	The heterogeneity was high in the retrospective studies and moderate in the prospective. The interventions differed due to different implant systems used. The implant type (rough surface, screw shaped) and the protocol for the placement were consistent	Very low
Indirectness	The studies were carried out in 6 different countries on 3 continents. They were conducted in university hospitals and specialist private practices	low
Imprecision	The sample size overall is small. The high number of patients LTFU was considered	Very low
Publication bias	The small number of studies makes the interpretation of funnel-plots difficult. However, the outliners balance each other out	low

### Data Synthesis

#### Primary Outcome

**Fig. 2:**
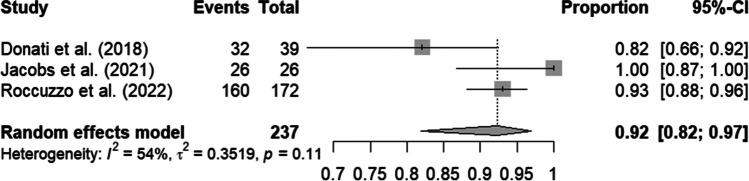
Forest plot: Complete case analysis of prospective studies. This only includes implants without missing data on the variable of interest

**Fig. 3:**
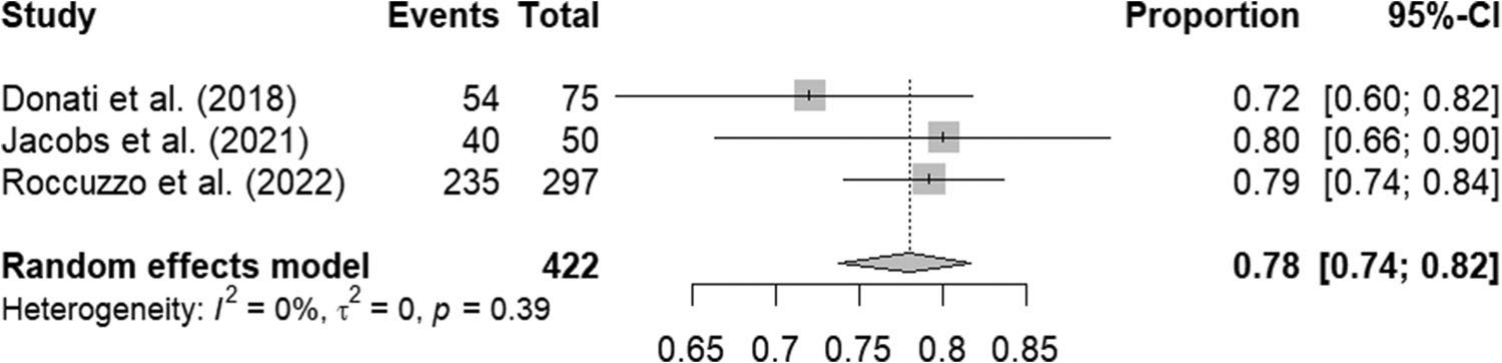
Forest plot: Imputed results. The proportion of failed implants is estimated to be five times higher in the lost-to-follow-up (LTFU) group than in the group that could be tracked

For the prospective studies, a complete-case analysis was conducted, resulting in a mean survival rate of 92% with a 95% CI of 82% to 97%. A total of 237 implants were included. There was a moderate level of heterogeneity at 54%, which was not statistically significant (p = 0.11) (Fig. 2). A best-case analysis is available in the supplementary documents. After imputation, the number of included implants increased to 422. The survival rate was significantly reduced to 78% (95% CI: 74%-82%). Heterogeneity was negligible at 0%, with p = 0.39 (Fig. 3).

**Fig. 4:**
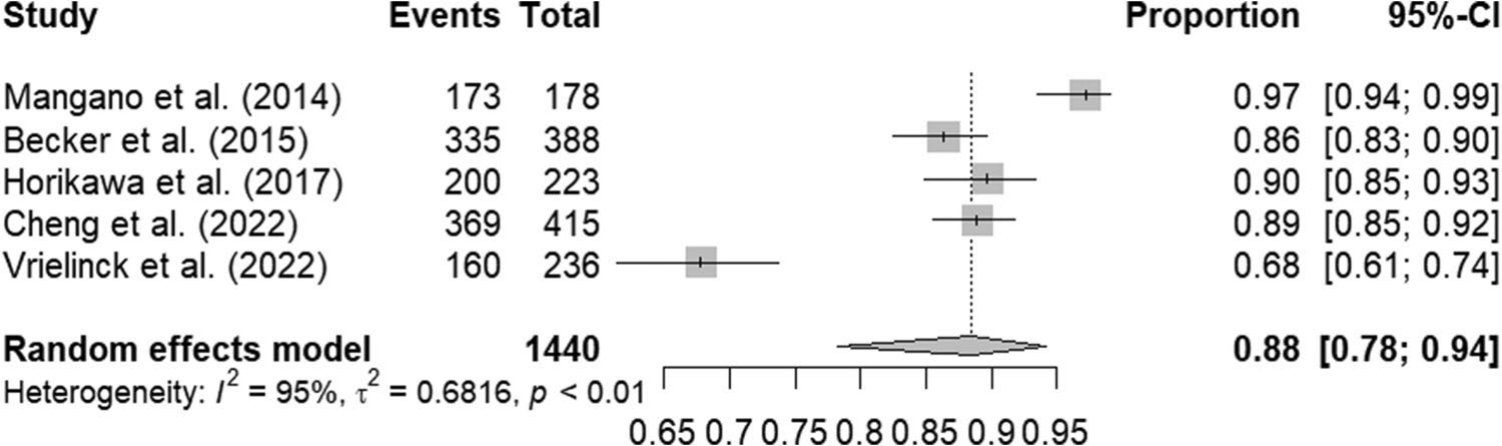
Forest plot: Analysis of retrospective studies

The retrospective studies included a total of 1440 implants and showed a survival rate of 88% (95% CI: 78%-94%) using Kaplan–Meier analysis. Heterogeneity was very high at 95% (p < 0.01) (Fig. 4).

Funnel plots of the analyses can all be found in the supplementary document, as the interpretive power is limited due to the small number of studies.

#### Descriptive results

The studies displayed heterogeneous approaches to data analysis and the variables considered. To provide insight into potential risk factors for implant survival, the following parameters with a significant impact on implant survival are summarized descriptively.

Whether failure was attributed to biological complications or fractures varied depending on the study. Donati recorded six fractures and one disintegration, while Roccuzzo recorded eleven losses due to peri-implantitis and one due to a fracture in a prosthetic restoration with a cantilever. Retrospective studies did not differentiate between these two causes or did not specify the reason for the lost implants.

In the study by Horikawa et al., Implant type, keratinized mucosa width (over 2mm), and gender were the only factors found to impact the prevalence of peri-implant infections [17]. They reported hazard ratios of 40.09 (p = 0.0012) for maxilla versus anterior mandible implants and 18.69 (p = 0.0013) for maxilla versus posterior mandible implants [17]. Cheng also reported higher survival rates for implants in the posterior mandible [23]. Regarding implant position Donati, Mangano, Becker and Jacobs had nearly equal distribution between mandible and maxilla but did not analyze differences in the prognosis. Vrielinck only placed implants in the anterior maxilla [22]. Single crowns were associated with a better prognosis and diabetes worsened the outcome [23]. Cheng also analyzed the differences between groups with osteoporosis. These groups were not included in our statistical analysis, only the healthy control group. However, it should be noted that adequate antiresorptive therapy (survival rate: oral: 94% (CI: 90–96%), injectible: 90% (CI: 78–97%)) can mitigate the increased risk of implant patients with osteoporosis/osteopenia (84% (CI: 79–88%)) [23]. Vrielinck et al. also reported a higher implant survival rate for fixed prostheses compared to removable restorations. Short implants and patients with bruxism had a higher likelihood of failure (with all losses occurring within the first month after implant placement) [22]. Becker identified smoking and implantation type (according to the ITI Consensus Conference) as significant factors [16]. Bone grafting was also not an exclusion criterion in our analysis. Among the included studies, only Becker et al. and Cheng et al. analyzed the difference between augmentation and no augmentation, and both found no significant differences [16, 23]. Conversely, Donati et al. and Roccuzzo et al. excluded cases with bone augmentation [18, 20]. Notably, in Cheng et al.'s study, bone augmentation in the osteoporotic group led to reduced survival rates.

Since many studies included periodontally compromised patients, radiological bone loss was occasionally noted. Donati observed an average bone loss of -0.83 mm (95% CI: -1.38/-0.28), with 34% showing no bone loss at all [18]. Jacobs only noted a loss of 0.13 +—0.34 mm (SD), ranging from -0.44 to 0.92mm [19]. Becker reported bone loss exceeding 2.5 mm in 18.5% of implants, and nearly 10% of implants showed signs of peri-implantitis [16, 25].

No study explicitly excluded patients with periodontal disease per se. In the case of Donati, patients had moderate or advanced periodontitis, yet only 5 implants exhibited signs of peri-implantitis (bleeding on probing/suppuration and bone loss exceeding 1 mm) [18]. Roccuzzo compared groups with different levels of periodontal disease, but found no differences in survival rates [20]. In contrast, Becker observed implant loss in 17 patients with periodontitis and only in 7 without [16]. Horikawa detected peri-implant infections in 48 implants, with an incidence of 21% after 15 years and 27.9% after 25 years [17].

## Discussion

### Strengths and relevance

For the very first time, this meta-analysis compiles data on the survival of dental implants after 20 years. The data are exclusively derived from more recent publications, which can be attributed to the fact that the technological advancements in dental implantology over the past decades have been significant [11, 12]. At present, the most commonly used format is the screw-shaped titanium implant with a rough surface, and thus, only these were included in the analysis [26].

When comparing the results to meta-analyses covering 5 and 10-year survival, it is evident that implant survival rates consistently remain well above 90% within these shorter time frames [3, 4, 27, 28]. This, however, does not guarantee a decrease in complication rates in the second decade during which the implant remains in function. This factor should be considered, particularly in light of increasing life expectancy, as it necessitates that more implants must remain functional for longer durations [29, 30]. Even when broadly comparing the results, the therapy involving dental implants can be regarded as a successful concept, especially when compared to total or unicondylar knee replacement, which show survival rates of 82% and 70% after 25 years [31]. It is also favorable when compared to total hip arthroplasty, which has a survival rate of only 60.4%-77.7% after 20 years [32].

Moreover, it is still open for discussion whether the prognosis of complex periodontal therapy may not compare favorably with the high success rates of implant treatment [33, 34]. Comparative and well-planned studies conducted over very long periods could provide valuable insights. Nevertheless, as demonstrated here, after 20 years, a non-negligible proportion of implants are lost. Therefore, the recommendation can only be that, as described by Pjetursson, implants should replace truly lost teeth and not the natural tooth itself [6].

This review was conducted in collaboration with statistically trained scientists and clinically experienced practitioners, thus providing a well-founded and practical source for both clinical practice and research [35]. Each individual study was scrutinized for statistical plausibility. Survival rates are a frequently requested parameter that holds high clinical significance in both medicine and dentistry [36]. To determine them, it is often necessary to employ statistical models that account for information loss due to increasing Lost to Follow-Up (LTFU) rates, with the Kaplan–Meier analysis being a particularly established method [37, 38]. While some previous works only included prospective studies, which, in principle, adhere to higher quality standards, they frequently did not employ Kaplan–Meier analysis, as is also the case with our example [3, 5, 39]. Therefore, we included retrospective works as they crucially contributed to achieving a sufficient sample size for this review. A pure complete case analysis often overestimates the success of a treatment [15]. To address data loss due to LTFU, we used an imputation method following Akl et al., which should be considered a rather conservative approach, as it likely overestimates the loss rate in the LTFU group [3, 14, 15]. No studies have addressed whether patients who were LTFU did not return for follow-ups because the implant was complication-free or because it was lost. However, a study by Lee et al. mentions that patients with poor compliance show a risk of tooth loss twice as high as the regular-compliance group [40]. The authors of the individual studies unanimously state that the reported data likely overestimate the survival rate. Our result shows that it is very likely to be the truth, and even if the imputed data may be too negative, the confidence intervals still overlap with those of the retrospective studies. It is substantiated to claim that approximately 4 out of 5 implants survive after 20 years.

When considering the extended timeframe, a sample size of 1440 plus 237 for this review is deemed sufficient [28, 39]. The most recent meta-analysis by Howe et al. included 2688 implants for the 10-year survival rate [3]. Previous works, depending on the research question, also encompassed between 101 and 1435 implants for the relatively short period of just 10 years [5, 41]. The long follow-up period is also extraordinary since in 2010 the median length of follow-up in RCTs was one year [42].

The literature search employed general terms and was supplemented by a search in the reference lists of the included publications, thus presenting a comprehensive literature view. All works are of recent date, reflecting the current state of treatment. They originate from six countries and three continents, providing a worldwide perspective [43]. This is particularly significant within systematic reviews in implantology, as, in the majority of cases, they involve single-center studies [42]. They were conducted in both university clinics and private practices, further enhancing external validity [43]. In absolute numbers, more implants are placed in private practice, while most studies are conducted in university clinics [44]. Therefore, the inclusion of data from both settings is particularly valuable. For example, Da Silva found a significantly lower survival rate after 5 years in private practice compared to data from university centers, but this may also be largely attributed to the pre-selected patient population [45].

### Limitations

In general, studies in dentistry and implantology face a common challenge, which is a high rate of patients lost to follow-up [42, 46]. This factor is particularly pronounced in prospective studies, as evident from the different results post-imputation, suggesting a very high risk of bias [47, 48]. This can be deemed sufficient for estimating the survival rate as a reference for clinical practice. Presenting it as "4 out of 5 implants survive after 20 years" aids patient comprehension, as opposed to abstract percentage figures [49].

The decision to include retrospective studies offers the advantages mentioned above but also comes with limitations. Retrospective data collection always carries the risk of data being inaccurately remembered or incompletely documented [50]. The inability to establish a causal relationship does not play a crucial role in the central question concerning the survival rate [50]. While retrospective studies conducted Kaplan–Meier analyses, this method of analysis was missing in prospective studies. Only three publications provided a 95% confidence interval, and one publication's data was deemed implausible, leading to its exclusion [46, 51].

It's worth noting that among the prospective studies, only one RCT was included in the analysis. While this may be a disadvantage for comparative analysis, it has little influence on the results of the survival rates. Furthermore, the external validity of cohort studies tends to be greater [50, 52].

One drawback in the statistical analysis across all studies was the failure to consider death as a competing risk factor. Such an analysis is applied in other fields, such as kidney transplantation [53]. Although the number of patients dropping out of the study due to death was partially recorded, there was a lack of corresponding implant numbers and consideration in the Kaplan–Meier analysis. Future studies in implantology need to account for this factor, considering that an aging population retains more implants beyond their lifespans [5]. In conclusion, it is suggested that, in light of increasing demands for study quality, dental implantology can best meet scientific standards by consulting with a statistically trained expert [54, 55].

Rather than a limitation of this meta-analysis, the lack of distinction between implant survival and success stems from missing studies. It cannot be emphasized enough that data based on a standardized definition of treatment success would be more insightful than survival rates alone.

### Descriptive results

In the present studies, various factors are mentioned that have a significant impact on implant survival. In general, they align with information from other literature and studies conducted over a shorter time frame. We will not discuss every single point extensively since systematic reviews are available in most cases.

The literature often states that the lower bone quality of the maxilla is mainly responsible for the lower survival rate in this region [56]. The low survival rate in Vrielinck’s study might also be attributed to the exclusive placement in the anterior maxilla. Horikawa's results are consistent with other authors who have also reported higher bone loss for implants placed in the maxilla [57]. Data on how diabetes affects implant survival have been heterogeneous. If anything, there is a tendency for a negative influence, but two meta-analyses could not find a significantly increased relative risk [58, 59]. A meta-analysis showed that osteoporosis does not necessarily represent a risk factor for implant loss. But a systematic review also sees a slight advantage regarding peri-implant bone loss with antiresorptive therapy. Adequate treatment of the underlying condition can be most likely derived from the existing data [60, 61]. A systematic review from 2016 sees no survival disadvantage for implants under 10mm and considers them equivalent to longer implants [62]. However, most of the currently used implants are 10mm or shorter. Another study that defines short implants as 5-6mm also sees comparable results for the 5–10-year survival rate as with longer implants. Emphasis is placed on the correct indication and application [63]. A systematic review by Kern et al. not only supports the aforementioned higher survival rate of implants in the mandible but also the better prognosis of fixed restorations as recorded by Vrielinck. The correct indication is also indispensable for this topic [64]. Consistent with the results of Cheng, Jung et al. also report outstanding survival rates for implants restored with single crowns at 96.3% (95% CI: 94.2–97.6%) [27]. In Vrielinck's study, losses occurred in patients with bruxism only in the first months after implantation, which matches the overall literature [65]. Therapy should be adapted (e.g., the number of implants: two instead of four) [22, 66]. Smoking is widely known as a risk factor for periodontitis and in implant therapy. Mustapha et al. also conclude that smokers have a 140.2% higher risk of implant failure [67]. Naseri has shown that the risk of loss also increases with the number of cigarettes smoked daily [68]. They thus support Becker's results. The ITI implantation type receives little attention in the literature, and reliable data are still lacking. A search on PubMed for the term "ITI implantation type" in titles and abstracts yielded only the study by Becker et al. as a result.

Even though periodontal diseases seem to be an important prognostic factor, as described by other authors, excluding these patients from this review is not meaningful due to the high prevalence in the population with over 40% of people affected in the US [69, 70]. Especially since Roccuzzo could not find significant differences in the survival rate of dental implants [20]. The inclusion and consideration of these patients contribute to the external validity of this study.

None of the studies recorded patient-related outcomes. Especially after such a long duration, patient satisfaction would have been of great interest and should be included in the future. Although it is a relatively new topic that only received increased attention in medical research in the 1980s and 2000s and challenges regarding validity still exist, they are essential to validate more patient-centered treatments in the future [71].

The risk factors recorded by the included studies largely represent results that are covered in other literature. This suggests that these studies provide realistic depictions of reality [43]. However, it must be noted that all factors in this review were identified by a maximum of one study. There was no consensus, but this can also be attributed to differing data and not all information being available. It can be assumed that the reasons for implant failure are multifactorial and cannot be attributed to a single cause or risk factor [72–74]. It will be important to closely follow up with patients and consistently treat comorbidities like osteoporosis, bruxism, or periodontitis over a long period, as the initial status may not necessarily persist after 20 years [22, 23]. To sum up, periodontitis, diabetes, and osteoporosis are conditions that should not necessarily contraindicate implant placement when adequately treated, as the long-term prognosis is, at most, only slightly reduced. Although only one study indicated a reduced survival rate associated with nicotine abuse, we strongly recommend smoking cessation to minimize potential complications. Some risk factors, such as unfavorable placement, may only show their negative effect after years, e.g., through implant fracture due to material fatigue [18]. Implant patients should not leave the practice without adequately planned follow-up after the operation. Continuous check-ups will be key to preventing complications by identifying risk factors or uncontrolled health issues.

## Conclusion

### So how far can we go?

For the first time, this review consolidates data on dental implant survival over a 20-year period. A survival rate of approximately 4 out of 5 implants is still considered remarkably good in the medical field for such a time frame. However, certain aspects have also emerged that will require further attention in the future. The significant difference in survival rates between 10 and 20 years indicates that dental implant therapy does not conclude after the initial surgery but also necessitates lifelong follow-up care. The research challenge ahead lies in pinpointing the pertinent risk factors within this timeframe and crafting strategies to ensure sustained implant survival, considering the likely multifactorial nature of implant failure. In this context, giving special consideration to quality standards is crucial to prevent overestimating the effectiveness of current treatments due to potential statistical errors.

High-quality and reliable therapies have been developed in dental implantology, but the conclusion remains: we can go even further.

## Supplementary Information

Below is the link to the electronic supplementary material.

Supplementary file1 (DOCX 1132 KB)

## Data Availability

No datasets were generated or analysed during the current study.

## References

[1] Gupta R, Gupta N, Weber, DDS KK (2023) Dental implants. In: StatPearls [Internet]. StatPearls Publishing, Treasure island (FL). Available from: https://www.ncbi.nlm.nih.gov/books/NBK470448/29262027

[2] Elani HW, Starr JR, Da Silva JD et al (2018) Trends in dental implant use in the U.S., 1999–2016, and projections to 2026. J Dent Res 97:1424–1430. 10.1177/0022034518792567 30075090 10.1177/0022034518792567 PMC6854267

[3] Howe M-S, Keys W, Richards D (2019) Long-term (10-year) dental implant survival: a systematic review and sensitivity meta-analysis. J Dent 84:9–21. 10.1016/j.jdent.2019.03.008 30904559 10.1016/j.jdent.2019.03.008

[4] Hjalmarsson L, Gheisarifar M, Jemt T (2016) A systematic review of survival of single implants as presented in longitudinal studies with a follow-up of at least 10 years. Eur J Oral Implantol 9(Suppl 1):S155–S162 27314122

[5] Srinivasan M, Meyer S, Mombelli A et al (2017) Dental implants in the elderly population: a systematic review and meta-analysis. Clin Oral Implants Res 28:920–930. 10.1111/clr.12898 27273468 10.1111/clr.12898

[6] Pjetursson BE, Heimisdottir K (2018) Dental implants - are they better than natural teeth? Eur J Oral Sci 126(Suppl 1):81–87. 10.1111/eos.12543 30178552 10.1111/eos.12543

[7] Torabinejad M, Goodacre CJ (2006) Endodontic or dental implant therapy: the factors affecting treatment planning. J Am Dent Assoc 137:973–7 quiz 1027–8. 10.14219/jada.archive.2006.0318 10.14219/jada.archive.2006.0318 16803823

[8] Tomasi C, Albouy J-P, Schaller D et al (2022) Efficacy of rehabilitation of stage IV periodontitis patients with full-arch fixed prostheses: Tooth-supported versus Implant-supported-A systematic review. J Clin Periodontol 49(Suppl 24):248–271. 10.1111/jcpe.13511 34761430 10.1111/jcpe.13511

[9] Lu P, Gong Y, Chen Y et al (2014) Safety analysis of tooth extraction in elderly patients with cardiovascular diseases. Med Sci Monit 20:782–788. 10.12659/MSM.890131 24819043 10.12659/MSM.890131 PMC4031223

[10] Gonçalves GSY, de Magalhães K, Ferreira M, Rocha EP et al (2022) Oral health-related quality of life and satisfaction in edentulous patients rehabilitated with implant-supported full dentures all-on-four concept: a systematic review. Clin Oral Investig 26:83–94. 10.1007/s00784-021-04213-y 34647147 10.1007/s00784-021-04213-y

[11] Bonanthaya K, Panneerselvam E, Manuel S et al (eds) (2021) Oral and Maxillofacial Surgery for the Clinician. Springer Nature Singapore, Singapore

[12] Ebenezer S, Kumar VV, Thor A (2021) Basics of Dental Implantology for the Oral Surgeon. In: Bonanthaya K, Panneerselvam E, Manuel S et al (eds) Oral and Maxillofacial Surgery for the Clinician. Springer Nature Singapore, Singapore, pp 385–405

[13] Hoy D, Brooks P, Woolf A et al (2012) Assessing risk of bias in prevalence studies: modification of an existing tool and evidence of interrater agreement. J Clin Epidemiol 65:934–939. 10.1016/j.jclinepi.2011.11.014 22742910 10.1016/j.jclinepi.2011.11.014

[14] Akl EA, Briel M, You JJ et al (2012) Potential impact on estimated treatment effects of information lost to follow-up in randomised controlled trials (LOST-IT): systematic review. BMJ 344:e2809. 10.1136/bmj.e2809 22611167 10.1136/bmj.e2809

[15] Akl EA, Johnston BC, Alonso-Coello P et al (2013) Addressing dichotomous data for participants excluded from trial analysis: a guide for systematic reviewers. PLoS ONE 8:e57132. 10.1371/journal.pone.0057132 23451162 10.1371/journal.pone.0057132 PMC3581575

[16] Becker ST, Beck-Broichsitter BE, Rossmann CM et al (2016) Long-term Survival of Straumann Dental Implants with TPS Surfaces: A Retrospective Study with a Follow-up of 12 to 23 Years. Clin Implant Dent Relat Res 18:480–488. 10.1111/cid.12334 25810237 10.1111/cid.12334

[17] Horikawa T, Odatsu T, Itoh T et al (2017) Retrospective cohort study of rough-surface titanium implants with at least 25 years’ function. Int J Implant Dent 3:42. 10.1186/s40729-017-0101-7 28875460 10.1186/s40729-017-0101-7 PMC5585117

[18] Donati M, Ekestubbe A, Lindhe J et al (2018) Marginal bone loss at implants with different surface characteristics - A 20-year follow-up of a randomized controlled clinical trial. Clin Oral Implants Res 29:480–487. 10.1111/clr.13145 29569767 10.1111/clr.13145

[19] Jacobs R, Gu Y, Quirynen M et al (2021) A 20-year split-mouth comparative study of two screw-shaped titanium implant systems. Int J Oral Implantol (Berl) 14:421–430 34726850

[20] Roccuzzo A, Imber J-C, Marruganti C et al (2022) Clinical outcomes of dental implants in patients with and without history of periodontitis: A 20-year prospective study. J Clin Periodontol 49:1346–1356. 10.1111/jcpe.13716 36054302 10.1111/jcpe.13716 PMC9804375

[21] Mangano C, Iaculli F, Piattelli A et al (2015) Fixed restorations supported by Morse-taper connection implants: a retrospective clinical study with 10–20 years of follow-up. Clin Oral Implants Res 26:1229–1236. 10.1111/clr.12439 24954285 10.1111/clr.12439

[22] Vrielinck L, Blok J, Politis C (2022) Survival of conventional dental implants in the edentulous atrophic maxilla in combination with zygomatic implants: a 20-year retrospective study. Int J Implant Dent 8:27. 10.1186/s40729-022-00425-3 35704150 10.1186/s40729-022-00425-3 PMC9200924

[23] Cheng Y-C, Ewers R, Morgan K et al (2022) Antiresorptive therapy and dental implant survival: an up to 20-year retrospective cohort study in women. Clin Oral Investig 26:6569–6582. 10.1007/s00784-022-04609-4 36001145 10.1007/s00784-022-04609-4

[24] Guyatt GH, Thorlund K, Oxman AD et al (2013) GRADE guidelines: 13. Preparing summary of findings tables and evidence profiles-continuous outcomes. J Clin Epidemiol 66:173–183. 10.1016/j.jclinepi.2012.08.001 23116689 10.1016/j.jclinepi.2012.08.001

[25] Ong CTT, Ivanovski S, Needleman IG et al (2008) Systematic review of implant outcomes in treated periodontitis subjects. J Clin Periodontol 35:438–462. 10.1111/j.1600-051X.2008.01207.x 18433385 10.1111/j.1600-051X.2008.01207.x

[26] Kim WJ, Cho Y-D, Ku Y et al (2022) The worldwide patent landscape of dental implant technology. Biomaterials Research 26:59. 10.1186/s40824-022-00307-0 36274171 10.1186/s40824-022-00307-0 PMC9590213

[27] Jung RE, Zembic A, Pjetursson BE et al (2012) Systematic review of the survival rate and the incidence of biological, technical, and aesthetic complications of single crowns on implants reported in longitudinal studies with a mean follow-up of 5 years. Clin Oral Implants Res 23(Suppl 6):2–21. 10.1111/j.1600-0501.2012.02547.x 23062124 10.1111/j.1600-0501.2012.02547.x

[28] Pjetursson BE, Thoma D, Jung R et al (2012) A systematic review of the survival and complication rates of implant-supported fixed dental prostheses (FDPs) after a mean observation period of at least 5 years. Clin Oral Implants Res 23(Suppl 6):22–38. 10.1111/j.1600-0501.2012.02546.x 23062125 10.1111/j.1600-0501.2012.02546.x

[29] Veena S Raleigh (2019) Trends in life expectancy in EU and other OECD countries. 10.1787/223159ab-en

[30] Becker W, Hujoel P, Becker BE et al (2016) Dental implants in an aged population: evaluation of periodontal health, bone loss, implant survival, and quality of life. Clin Implant Dent Relat Res 18:473–479. 10.1111/cid.12340 26082299 10.1111/cid.12340

[31] Evans JT, Walker RW, Evans JP et al (2019) How long does a knee replacement last? a systematic review and meta-analysis of case series and national registry reports with more than 15 years of follow-up. Lancet 393:655–663. 10.1016/S0140-6736(18)32531-5 30782341 10.1016/S0140-6736(18)32531-5 PMC6381229

[32] Negm AM, Beaupre LA, Goplen CM et al (2022) A Scoping review of total hip arthroplasty survival and reoperation rates in patients of 55 years or younger: health services implications for revision surgeries. Arthroplast Today 16:247-258.e6. 10.1016/j.artd.2022.05.012 36092132 10.1016/j.artd.2022.05.012 PMC9458900

[33] Rasperini G, Siciliano VI, Cafiero C et al (2014) Crestal bone changes at teeth and implants in periodontally healthy and periodontally compromised patients. A 10-year comparative case-series study. J Periodontol 85:e152–e159. 10.1902/jop.2013.130415 24215202 10.1902/jop.2013.130415

[34] Lang NP (2019) Oral implants: the paradigm shift in restorative Dentistry. J Dent Res 98:1287–1293. 10.1177/0022034519853574 31633460 10.1177/0022034519853574

[35] Berman NG, Parker RA (2002) Meta-analysis: neither quick nor easy. BMC Med Res Methodol 2:10. 10.1186/1471-2288-2-10 12171604 10.1186/1471-2288-2-10 PMC122061

[36] Shreffler J, Huecker MR (2023) Survival analysis. In: StatPearls [Internet]. StatPearls Publishing, Treasure Island (FL). Available from: https://www.ncbi.nlm.nih.gov/books/NBK560604/32809439

[37] Singh R, Mukhopadhyay K (2011) Survival analysis in clinical trials: Basics and must know areas. Perspect Clin Res 2:145–148. 10.4103/2229-3485.86872 22145125 10.4103/2229-3485.86872 PMC3227332

[38] Goel MK, Khanna P, Kishore J (2010) Understanding survival analysis: Kaplan-Meier estimate. Int J Ayurveda Res 1:274–278. 10.4103/0974-7788.76794 21455458 10.4103/0974-7788.76794 PMC3059453

[39] Papaspyridakos P, Mokti M, Chen C-J et al (2014) Implant and prosthodontic survival rates with implant fixed complete dental prostheses in the edentulous mandible after at least 5 years: a systematic review. Clin Implant Dent Relat Res 16:705–717. 10.1111/cid.12036 23311617 10.1111/cid.12036

[40] Lee CT, Huang HY, Sun TC et al (2015) Impact of Patient Compliance on Tooth Loss during Supportive Periodontal Therapy: A Systematic Review and Meta-analysis. J Dent Res 94:777–786. 10.1177/0022034515578910 25818586 10.1177/0022034515578910

[41] Moraschini V, da Poubel LA, C, Ferreira VF, et al (2015) Evaluation of survival and success rates of dental implants reported in longitudinal studies with a follow-up period of at least 10 years: a systematic review. Int J Oral Maxillofac Surg 44:377–388. 10.1016/j.ijom.2014.10.023 25467739 10.1016/j.ijom.2014.10.023

[42] Cairo F, Sanz I, Matesanz P et al (2012) Quality of reporting of randomized clinical trials in implant dentistry. A systematic review on critical aspects in design, outcome assessment and clinical relevance. J Clin Periodontol 39(Suppl 12):81–107. 10.1111/j.1600-051X.2011.01839.x 10.1111/j.1600-051X.2011.01839.x 22533949

[43] Rothwell PM (2006) Factors that can affect the external validity of randomised controlled trials. PLoS Clin Trials 1:e9. 10.1371/journal.pctr.0010009 16871331 10.1371/journal.pctr.0010009 PMC1488890

[44] Kourtis SG, Sotiriadou S, Voliotis S et al (2004) Private practice results of dental implants. Part I: survival and evaluation of risk factors–Part II: surgical and prosthetic complications. Implant Dent 13:373–385. 10.1097/01.id.0000148564.88384.de 15592000 10.1097/01.id.0000148564.88384.de

[45] Da Silva JD, Kazimiroff J, Papas A et al (2014) Outcomes of implants and restorations placed in general dental practices: a retrospective study by the Practitioners Engaged in Applied Research and Learning (PEARL) Network. J Am Dent Assoc 145:704–713. 10.14219/jada.2014.27 24982276 10.14219/jada.2014.27 PMC5266561

[46] Meijer HJA, Raghoebar GM (2012) Quality of reporting of descriptive studies in implant dentistry. Critical aspects in design, outcome assessment and clinical relevance. J Clin Periodontol 39 Suppl 12:108–113. 10.1111/j.1600-051X.2011.01834.x 10.1111/j.1600-051X.2011.01834.x 22533950

[47] Boutron I, Moher D, Altman DG et al (2008) Methods and processes of the CONSORT group: example of an extension for trials assessing nonpharmacologic treatments. Ann Intern Med 148:W60–W66. 10.7326/0003-4819-148-4-200802190-00008-w1 18283201 10.7326/0003-4819-148-4-200802190-00008-w1

[48] Robins JM, Rotnitzky A, Scharfstein DO (2000) Sensitivity Analysis for Selection bias and unmeasured Confounding in missing Data and Causal inference models. In: Halloran ME, Berry D (eds) Statistical Models in Epidemiology, the Environment, and Clinical Trials. Springer, New York, New York, NY, pp 1–94

[49] Fagerlin A, Zikmund-Fisher BJ, Ubel PA (2011) Helping patients decide: ten steps to better risk communication. J Natl Cancer Inst 103:1436–1443. 10.1093/jnci/djr318 21931068 10.1093/jnci/djr318 PMC3218625

[50] Euser AM, Zoccali C, Jager KJ et al (2009) Cohort studies: prospective versus retrospective. Nephron Clin Pract 113:c214–c217. 10.1159/000235241 19690438 10.1159/000235241

[51] Park W-B, Kang KL, Han J-Y (2019) Factors influencing long-term survival rates of implants placed simultaneously with lateral maxillary sinus floor augmentation: a 6- to 20-year retrospective study. Clin Oral Implants Res 30:977–988. 10.1111/clr.13505 31306519 10.1111/clr.13505

[52] Booth CM, Tannock IF (2014) Randomised controlled trials and population-based observational research: partners in the evolution of medical evidence. Br J Cancer 110:551–555. 10.1038/bjc.2013.725 24495873 10.1038/bjc.2013.725 PMC3915111

[53] Pinto-Ramirez J, Garcia-Lopez A, Salcedo-Herrera S et al (2022) Risk factors for graft loss and death among kidney transplant recipients: A competing risk analysis. PLoS ONE 17:e0269990. 10.1371/journal.pone.0269990 35834500 10.1371/journal.pone.0269990 PMC9282472

[54] Bhatavadekar N (2010) Helping the clinician make evidence-based implant selections. A systematic review and qualitative analysis of dental implant studies over a 20 year period. Int Dent J 60:359–369 21141209

[55] Faggion CM, JR, Schmitter M, (2010) Using the best available evidence to support clinical decisions in implant dentistry. Int J Oral Maxillofac Implants 25:960–969 20862410

[56] Ko Y-C, Huang H-L, Shen Y-W et al (2017) Variations in crestal cortical bone thickness at dental implant sites in different regions of the jawbone. Clin Implant Dent Relat Res 19:440–446. 10.1111/cid.12468 28074591 10.1111/cid.12468

[57] Moraschini V, Barboza EdP (2016) Success of dental implants in smokers and non-smokers: a systematic review and meta-analysis. Int J Oral Maxillofac Surg 45:205–215. 10.1016/j.ijom.2015.08.996 26385308 10.1016/j.ijom.2015.08.996

[58] Moraschini V, Barboza ESP, Peixoto GA (2016) The impact of diabetes on dental implant failure: a systematic review and meta-analysis. Int J Oral Maxillofac Surg 45:1237–1245. 10.1016/j.ijom.2016.05.019 27297836 10.1016/j.ijom.2016.05.019

[59] Souto-Maior JR, Pellizzer EP, de Luna Gomes, Jéssica Marcela, et al (2019) Influence of diabetes on the survival rate and marginal bone loss of dental implants: an overview of systematic reviews. J Oral Implantol 45:334–340. 10.1563/aaid-joi-D-19-00087 31042455 10.1563/aaid-joi-D-19-00087

[60] de Medeiros FCFL, Kudo GAH, Leme BG et al (2018) Dental implants in patients with osteoporosis: a systematic review with meta-analysis. Int J Oral Maxillofac Surg 47:480–491. 10.1016/j.ijom.2017.05.021 28651805 10.1016/j.ijom.2017.05.021

[61] Fiorillo L, Cicciù M, Tözüm TF et al (2022) Impact of bisphosphonate drugs on dental implant healing and peri-implant hard and soft tissues: a systematic review. BMC Oral Health 22:291. 10.1186/s12903-022-02330-y 35843929 10.1186/s12903-022-02330-y PMC9288700

[62] Sierra-Sánchez J-L, García-Sala-Bonmatí F, Martínez-González A et al (2016) Predictability of short implants (< 10 mm) as a treatment option for the rehabilitation of atrophic maxillae. A systematic review. Med Oral Patol Oral Cir Bucal 21:e392-402. 10.4317/medoral.20949 26946199 10.4317/medoral.20949 PMC4867215

[63] Rameh S, Menhall A, Younes R (2020) Key factors influencing short implant success. Oral Maxillofac Surg 24:263–275. 10.1007/s10006-020-00841-y 32323043 10.1007/s10006-020-00841-y

[64] Kern J-S, Kern T, Wolfart S et al (2016) A systematic review and meta-analysis of removable and fixed implant-supported prostheses in edentulous jaws: post-loading implant loss. Clin Oral Implants Res 27:174–195. 10.1111/clr.12531 25664612 10.1111/clr.12531 PMC5024059

[65] Zhou Y, Gao J, Luo Le et al (2016) Does bruxism contribute to dental implant failure? a systematic review and meta-analysis. Clin Implant Dent Relat Res 18:410–420. 10.1111/cid.12300 25726844 10.1111/cid.12300

[66] Wen H, Guo W, Liang R et al (2014) Finite element analysis of three zygomatic implant techniques for the severely atrophic edentulous maxilla. J Prosthet Dent 111:203–215. 10.1016/j.prosdent.2013.05.004 24314571 10.1016/j.prosdent.2013.05.004

[67] Mustapha AD, Salame Z, Chrcanovic BR (2021) Smoking and dental implants: a systematic review and meta-analysis. Medicina (Kaunas) 58. 10.3390/medicina58010039 10.3390/medicina58010039 PMC8780868 35056347

[68] Naseri R, Yaghini J, Feizi A (2020) Levels of smoking and dental implants failure: A systematic review and meta-analysis. J Clin Periodontol 47:518–528. 10.1111/jcpe.13257 31955453 10.1111/jcpe.13257

[69] Kwon T, Lamster IB, Levin L (2021) Current concepts in the management of periodontitis. Int Dent J 71:462–476. 10.1111/idj.12630 34839889 10.1111/idj.12630 PMC9275292

[70] Carra MC, Rangé H, Swerts P-J et al (2022) Effectiveness of implant-supported fixed partial denture in patients with history of periodontitis: A systematic review and meta-analysis. J Clin Periodontol 49(Suppl 24):208–223. 10.1111/jcpe.13481 34775625 10.1111/jcpe.13481

[71] Churruca K, Pomare C, Ellis LA et al (2021) Patient-reported outcome measures (PROMs): a review of generic and condition-specific measures and a discussion of trends and issues. Health Expect 24:1015–1024. 10.1111/hex.13254 33949755 10.1111/hex.13254 PMC8369118

[72] Alvim-Pereira F, Montes CC, Thomé G et al (2008) Analysis of association of clinical aspects and vitamin D receptor gene polymorphism with dental implant loss. Clin Oral Implants Res 19:786–795. 10.1111/j.1600-0501.2008.01532.x 18705810 10.1111/j.1600-0501.2008.01532.x

[73] French D, Tallarico M (2014) Eight-year clinical and radiologic results of maxillary and mandibular implant-retained bar overdentures carried out on oxidized (TiUnite™) replace select implants placed in regenerated bone: a clinical case. Quintessence Int 45:135–140. 10.3290/j.qi.a31012 24389566 10.3290/j.qi.a31012

[74] Martin W, Lewis E, Nicol A (2009) Local risk factors for implant therapy. Int J Oral Maxillofac Implants 24(Suppl):28–38 19885433

